# Gene expression profiling for molecular distinction and characterization of laser captured primary lung cancers

**DOI:** 10.1186/1479-5876-6-69

**Published:** 2008-11-07

**Authors:** Astrid Rohrbeck, Judith Neukirchen, Michael Rosskopf, Guillermo G Pardillos, Helene Geddert, Andreas Schwalen, Helmut E Gabbert, Arndt von Haeseler, Gerald Pitschke, Matthias Schott, Ralf Kronenwett, Rainer Haas, Ulrich-Peter Rohr

**Affiliations:** 1 Department of Hematology, Oncology and Clinical Immunology, Heinrich-Heine-University Duesseldorf, Moorenstraße 5, 40225 Duesseldorf, Germany; 2 Institute for Bioinformatics, Heinrich-Heine-University Duesseldorf, Germany; 3 Department of Pathology, Heinrich-Heine-University Duesseldorf, Germany; 4 Department of Cardiology, Pneumology and Angiology, Heinrich-Heine-University Düsseldorf, Germany; 5 Center for Integrative Bioinformatics, Max F. Perutz Laboratories; University of Vienna; Medical University of Vienna; University of Veterinary Medicine Vienna, Vienna, Austria; 6 Department of Endocrinology, Diabetology and Rheumatology, Heinrich-Heine-University Düsseldorf, Germany; 7 Department of Hematology and Oncology, Innere Klinik I, Albert-Ludwigs-Universität Freiburg, Hugstetter Str. 55, 79106 Freiburg, Germany

## Abstract

**Methods:**

We examined gene expression profiles of tumor cells from 29 untreated patients with lung cancer (10 adenocarcinomas (AC), 10 squamous cell carcinomas (SCC), and 9 small cell lung cancer (SCLC)) in comparison to 5 samples of normal lung tissue (NT). The European and American methodological quality guidelines for microarray experiments were followed, including the stipulated use of laser capture microdissection for separation and purification of the lung cancer tumor cells from surrounding tissue.

**Results:**

Based on differentially expressed genes, different lung cancer samples could be distinguished from each other and from normal lung tissue using hierarchical clustering. Comparing AC, SCC and SCLC with NT, we found 205, 335 and 404 genes, respectively, that were at least 2-fold differentially expressed (estimated false discovery rate: < 2.6%). Different lung cancer subtypes had distinct molecular phenotypes, which also reflected their biological characteristics. Differentially expressed genes in human lung tumors which may be of relevance in the respective lung cancer subtypes were corroborated by quantitative real-time PCR.

Genetic programming (GP) was performed to construct a classifier for distinguishing between AC, SCC, SCLC, and NT. Forty genes, that could be used to correctly classify the tumor or NT samples, have been identified. In addition, all samples from an independent test set of 13 further tumors (AC or SCC) were also correctly classified.

**Conclusion:**

The data from this research identified potential candidate genes which could be used as the basis for the development of diagnostic tools and lung tumor type-specific targeted therapies.

## Background

Lung cancer represents a heterogeneous group of diseases in terms of their biology and the clinical course. The diagnosis and classification of lung cancers are primarily based on the histological morphology and immunohistological methods for distinguishing between small cell lung cancer (SCLC) and non-small cell lung cancer (NSCLC) [1]. The molecular pathogenesis of lung cancer, as far as it has been deciphered, consists of genetic and epigenetic alterations, including the activation of proto-oncogenes and inactivation of tumor suppressor genes [2-4]. This leads to a malignant phenotype, resulting in changes in cell structure, adhesion and cell proliferation [5].

Oligonucleotide microarray studies are commonly used to extend the knowledge of the differences in the biology of lung tumors and to identify new candidate genes with diagnostic, prognostic and therapeutic value [6-9]. Several gene expression profiling studies in lung cancer have been published, however, it is still difficult to compare these studies due to the differences in methodologies, array platforms, normalization of the data and biostatistical analyses approaches, which may influence the reproducibility and comparability [10-12]. Such differences could have led to divergent results, with limited overlap of described genes.

Another crucial step in the field of oligonucleotide microarray studies is the preparation of the solid tumor sample itself. It contains a variable amount of mesenchymal stroma cells, blood vessels, fibroblasts, tumor-invading lymphocytes and necrotic areas next to the tumor cells themselves. Analyzing the complete tumor sample without efficient separation of the tumor cell confounds the true gene expression profile of the tumor.

In order to overcome these methodological limitations, we followed the guidelines from the Microarray Gene Expression Data Society [13] and the MicroArray Quality Control (MAQC) Consortium [14,15], the External RNA Controls Consortium (ERCC) [16] as well as the European consensus guidelines for gene expression experiments [17]. The purification of the tumor cells was carried out by laser capture microdissection (LCM), which has been shown to greatly improve the sample preparation for microarray expression analysis [18]. Few reports on LCM and microarray gene expression analysis have been published to date, comparing all distinct lung cancer subtypes to normal lung tissue [19-21].

In this report, we performed a comparison of gene expression profiles, using microarray analysis and LCM, according to the methodological quality consensus guidelines for microarray experiments, with the aim of identifying genes that are differentially expressed in the major histological lung cancer subtypes, as compared to normal lung tissue. In addition, 14 differentially expressed genes in human lung tumors were corroborated by quantitative real-time PCR. Furthermore, using genetic programming, we found a subset of 40 genes, that could be utilized for the classification of different types of lung tumors.

## Materials and methods

### Lung tumor samples

Samples of lung tumors were obtained using bronchoscopy or CT-guided needle aspiration from 29 patients, newly diagnosed patients with lung cancer. The samples that were immediately fixed in RNA-later consisted of 10 adenocarcinomas, 10 squamous cell carcinomas and 9 small cell lung carcinomas. Control samples of normal lung tissue were obtained from 5 patients with suspected tuberculosis or sarcoidosis, without presence of malignant lung tumors. The histopathological diagnosis was based on routinely processed hematoxylin-eosin stains and confirmed by immunohistochemical staining looking for pan-cytokeratin, cytokeratin 5 and 7, chromogranin A, synaptophysin and tissue-transcripion-factor-1. For validation of the classificator from genetic programming, 13 lung cancer samples were selected as a test-set from patients with advanced NSCLC lung cancers. All patients gave their informed consent and the study was approved by the ethics committee of the Heinrich-Heine University, Duesseldorf.

### Laser capture microdissection

From each frozen tumor tissue, we prepared 8-μm thick sections. The sections were fixed in methanol/acetic acid and stained in hematoxylin. The tumor cells were identified and ascertained in the sample by an experienced pathologist using the Autopix 100 automated LCM system and collected on a CapSure HS LCM Cap (Arcturus, Mount View, CA). Following microdissection, total RNA-extraction was performed with the RNeasy Micro Kit (QIAamp DNA MicroKit Qiagen, Santa Clarita, CA, USA), according to the manufacturer's instruction. A standard quality control of the total RNA was performed using the Agilent 2100 Bioanalyzer (Agilent Technologies, Palo Alto, USA).

### RNA isolation, cRNA labeling and hybridization to microarrays

The described procedures strictly adhered to the guidelines from the Microarray Gene Expression Data Society and the MicroArray Quality Control (MAQC) Consortium, the External RNA Controls Consortium (ERCC), as well as the European consensus guidelines for gene expression experiments [13-17]. The full description of the Extraction protocol, labeling and labeling protocol, hybridization protocol and data processing is obtainable in the GEO DATA base under  (accession number GSE6044). Total RNA (median: 375 ng; range: 250 – 500 ng) was used to generate biotin-labeled cRNA (median: 6,5 μg; range: 3–10 μg) by means of Message Amp aRNA Amplification Kit (Ambion, Austin, TX). Quality control of RNA and cRNA was performed using a bioanalyzer (Agilent 2001 Biosizing, Agilent Technologies). Following fragmentation, labeled cRNA of each individual patient sample was hybridized to Affymetrix HG-Focus GeneChips, covering 8793 genes, and stained according to the manufacturer's instructions.

### Quantification, normalization and statistical analysis

The quality control, normalization and data analysis, were assured with the *affy *package of functions of statistical scripting language 'R' integrated into the Bioconductor project , as described previously [22]. Using histograms of perfect match intensities, 5' to 3' RNA degradation side-by-side plots, or scatter plots, we estimated the quality of samples and hybridizations. To normalize raw data, we used a method of variance stabilizing transformations (VSN) [23]. To compare the normalized data from AC, SCC, SCLC and normal lung tissue, we used the Significance Analysis of Microarrays (SAM) algorithm v2.23  which contains a sliding scale for false discovery rate (FDR) of significantly up- and downregulated genes [24]. All data were permuted 1000 times by using the two classes, unpaired data mode of the algorithm. As a cut-off for significance, an estimated FDR of 2.6% was chosen by the tuning parameter delta of the software. The significance level of each gene was given by the q-value describing the lowest FDR in multiple testing [25], and a cut-off for fold-change of differential expression of 2 was used.

Hierarchical clustering analysis (HCA) was used to determine components of variation in the data in this study. For these analyses we used the unsupervised complete linkage algorithm.

The data points were organized in a phylogenetic tree with the branch lengths represent the degree of similarity between the values. Significantly expressed genes were uploaded to KEGG (Kyoto Encyclopedia of Genes and Genomes) and functional annotation was performed. Genes that were not listed or could be classified in more than one functional group were reviewed for the function based on the literature available using Pubmed, OMIM and GENE available in .

### Quantitative real-time PCR

Corroboration of RNA expression data was performed by realtime PCR using the ABI PRISM 7900 HT Sequence Detection System Instrument (Applied Biosystems, Applera Deutschland GmbH, Darmstadt, Germany). Total RNA, ranging between 600 – 1000 ng, underwent reverse transcription using a High capacity cDNA Archive Kit according to the manufacturer's instruction (Applied Biosystems, Applera Deutschland GmbH, Darmstadt, Germany). PCRs were performed according to the instructions of the manufacturer, using commercially available assays-on-demand (Applied Biosystems, Applera Deutschland GmbH, Darmstadt, Germany). Ct values were calculated by the ABI PRISM software, and relative gene expression levels were expressed as the difference in Ct values of the target gene and the control gene ribosomal protein S11(RPS11). RPS11 was selected as reference gene for the quantification analyses, because the expression levels of the gene were similar between the examined tumor samples and normal tissue.

### Classification using genetic programming

In order to generate a classifier that distinguishes between AC, SCC and SCLC, as well as the normal lung tissue, a Genetic Programming (GP) approach was used. The software DISCIPULUS which implements GP [26] was utilized. A leave-one-out cross validation (LOOCV) was performed, whereby one sample is removed from the training set. The other samples are reduced to those 50 genes with the highest signal-to-noise ratio, which are used as a training set in a training series. A training series generates a number of classifiers. After each series, the 30 best resulting classifiers are applied to that sample removed before, and the number of exact predictions were counted. The procedure was iterated, so that every sample was outside the training set once. The percentages of exact predictions for all samples of a class using 1020 classifiers (34 tissue samples and 30 classifiers = 34 * 30 = 1020 classifiers) were calculated. Each classifier used 50 different genes of a sample, queried their expression values and made the decision of "part of the class" or "outside the class". For each classifier and LOOCV iteration, the frequency of a gene (how often a gene occurs as appropriate classifier) was determined. The frequency was used as a quality criterion. The 10 genes with the highest frequency in each of the four classes were chosen in order to generate a final classifier of 40 genes. The accuracy of correct classification of the tissue is calculated as percentage using 30 classifiers of all left-out samples.

## Results

### Expression profiles and hierarchical cluster analysis

In this study, we examined gene expression profiles of untreated tumor cells from 29 patients with lung cancer (10 adenocarcinomas, 10 squamous cell carcinomas, 9 small cell lung cancer) in comparison to 5 normal lung tissues. The original data set and the patients characteristics are available in the GEO DATA base under  (accession number GSE6044).

**Figure 1:**
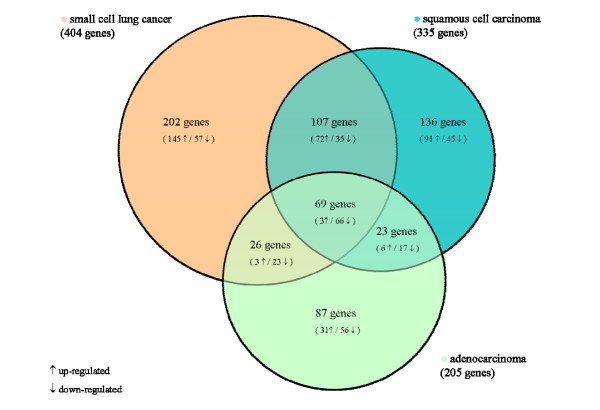
Venn Diagramm of significantly regulated genes comparing adenocarcinomas, squamous cell carcinomas and small cell lung cancer to normal lung tissue (NT). The 3 genes that were overexpressed in all 3 tumor types were chromosome condensation protein G (overexpression in AC vs. NT, SCC vs. NT and SCLC vs. NT was 2.2, 2.1 and 3.2-fold, respectively); collagen, type I, alpha 1 (overexpression in AC vs. NT, SCC vs. NT and SCLC vs. NT was 7.98, 3.24 and 2.4-fold, respectively) and mesoderm specific transcript homolog (overexpression in AC vs. NT, SCC vs. NT and SCLC vs. NT was 2.9, 4.5 and 14.8-fold, respectively).

**Figure 2:**
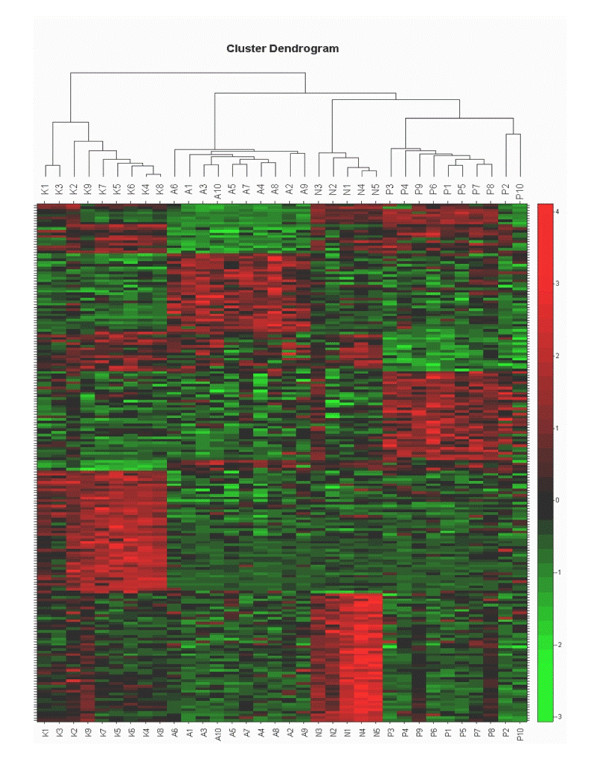
Consensus tree of hierarchical clustering of AC (A1–A10), SCC (P1–P10), SCLC (K1–K10) and lung tissue samples (NT) as a control without cancer (N1–N5) using the genes with the higherst differential expression according to the fold change from the comparison of AC vs. NT, SCC vs. NT and SCLC vs. NT. Data are displayed by a color code. Green, transcript levels below the median; black, equal to the median; red, greater than median. The effective length of the dash after sample separation visualizes the degree of similarity of the different samples.

Comparing AC, SCC and SCLC to normal lung tissue using significance analysis of microarrays (SAM), we found 205, 335 and 404 genes with an at least 2-fold different expression level and an estimated false discovery rate (FDR) of <2.6%. For an overview, a Venn diagram shows the overlaps of the three among groups (Figure 1) and the differentially expressed genes were further grouped in 14 functional classes (Table 1). Following SAM analysis, an unsupervised complete linkage clustering algorithm for cluster analyses was performed. The closest pair of the highest expression values of 198 differentially expressed genes was grouped together and a clear segregation of the analyzed groups (adenocarcinomas, squamous cell carcinomas, small cell lung cancer and normal lung tissue) was obtained (Figure 2)

**Table 1:** Classification of significantly deregulated lung cancer genes in comparison to normal lung tissue with respect to cancer subtype and biological functions.

functional class	Adenocarcinoma n = 205		squamous cell carcinoma n = 335		small cell lung cancer n = 404	
proliferation	1 gene ↑	1 gene ↓	41 genes ↑		56 genes ↑	1 gene ↓

DNA-repair		1 gene ↓	8 genes ↑		14 genes ↑	

oncogenes/tumor related genes	1 gene ↑	11 genes ↓	9 genes ↑	11 genes ↓	13 genes ↑	10 genes ↓

cell adhesion	3 genes ↑	6 genes ↓	2 genes ↑	6 genes ↓	8 genes ↑	7 genes ↓

cell structure	7 genes ↑	20 genes ↓	14 genes ↑	15 genes ↓	19 genes ↑	18 genes ↓

metabolism	11 genes ↑	25 genes ↓	34 genes ↑	26 genes ↓	22 genes ↑	38 genes ↓

immune system	6 genes ↑	13 genes ↓	3 genes ↑	19 genes ↓	2 genes ↑	25 genes ↓

signal transduction		13 genes ↓	7 genes ↑	3 genes ↓	18 genes ↑	10 genes ↓

transcription	1 gene ↑	8 genes ↓	10 genes ↑	5 genes ↓	22 genes ↑	4 genes ↓

transport	2 genes ↑	12 genes ↓	11 genes ↑	15 genes ↓	8 genes ↑	15 genes ↓

development	1 gene ↑	9 genes ↓	5 genes ↑	6 genes ↓	12 genes ↑	4 genes ↓

calcium-binding	3 genes ↑	4 genes ↓		3 genes ↓		4 genes ↓

apoptosis		1 gene ↓			3 genes ↑	1 gene ↓

unknown	7 genes ↑	38 genes ↓	28 genes ↑	54 genes ↓	26 genes ↑	44 genes ↓

### Adenocarcinomas

We found 205 deregulated genes in AC; 43 were upregulated and 162 were downregulated. Looking at oncogenes and tumor-associated genes, only the paraneoplastic antigen MA2 gene was upregulated. Focusing on genes involved in cell structure, 7 genes were upregulated 2 to 7.9-fold, compared to normal lung tissue. Next to the intermediary filament keratin 7 gene, 3 genes were involved in the actin metabolism such as thymosin beta-10, actin-related protein 2/3 complex subunit 1B and plastin 3. Four downregulated genes, involved in cell structure, were found in AC compared to normal lung tissue. These genes were tubulin alpha 3 and beta 2 involved in the assembly of microtubules and intermediary filaments, as well as keratin 5 and 15.

We also looked for genes involved in cell adhesion and migration and found integrin alpha 3, integrin beta 2 and intercellular adhesion molecule 1 to be upregulated in adenocarcinomas, while 6 genes, including the desmosomal cadherins desmoglein 3 and desmocollin 3, were significantly downregulated compared to normal lung tissue.

Examining the genes involved in cell cycle control and proliferation, we found only 2 genes differentially expressed. The chromosome condensation protein G was upregulated, while cyclin A1 was downregulated in comparison to normal lung tissue. Looking at genes involved in DNA repair, only the DNA mismatch repair gene mutS homolog 3 was downregulated (Table 2).

**Table 2:** Selection of significantly differentially expressed genes in adenocarcinomas focusing on cell structure, cell adhesion and oncogenesis.

**Gene Symbol**	**Gene Name**	**Fold Change** **AC vs. NT**	**q-value**
**cell structure**			

COL1A1	collagen, type I, alpha 1	7.98	1.22

KRT7	keratin 7	5.11	0.53

PLS3	plastin 3	2.43	0.53

TMSB10	thymosin, beta 10	2.01	0.89

ARPC1B	actin related protein 2/3 complex, 1B	2.38	1.22

TUBA3	tubulin, alpha 3	0.39	1.22

TUBB2	tubulin, beta 2	0.33	0.53

KRT15	keratin 15	0.06	0.53

KRT5	keratin 5	0.05	0.53

**cell adhesion**			

ICAM1	intercellular adhesion molecule 1	5.80	0.89

ITGB2	integrin, ί-2	2.48	1.22

ITGA3	integrin α-3	2.02	1.53

DSG3	desmoglein 3	0.48	0.53

DSC3	desmocollin 3	0.32	0.53

**oncogenesis**			

SERPINH1	serine (or cysteine) proteinase inhibitor, clade H	3.27	0.89

PNMA2	paraneoplastic antigen MA2	3.13	0.89

MSH3	mutS homolog 3	0.44	0.53

MYB	v-myb Avian Myeloblastosis viral oncogene homolog	0.39	0.53

RABL2B	rab-like 2B	0.34	0.53

RABL2A	rab-like 2A	0.27	0.53

AC = adenocarcinoma, NT = normal lung tissue

### Squamous cell carcinomas

In SCC, we found 335 deregulated genes, including 172 upregulated and 163 downregulated genes. Looking at oncogenes and tumor-associated genes, 4 genes of the RAS associated gene family; the oncogenes v-myc myelocytomatosis viral oncogene homolog, v-maf musculoaponeurotic fibrosarcoma oncogene homolog and pituitary tumor-transforming 1 were upregulated. Examining genes involved in cell structure and cell adhesion, we found 5 types of collagen genes, in particular the genes encoding for collagen type I alpha-1 and 2, type V alpha-2, type VI alpha-3 and type XI alpha-1 to be upregulated in comparison to normal lung tissue. Further, gap junction protein alpha 1 (43 kDa), a member of the connexin gene family and neuronal cell adhesion molecule, a member of the immunoglobulin superfamily were upregulated, while 6 other genes involved in cell adhesion such as the tight junction protein 3 and claudin 10 were downregulated in comparison to normal lung tissue. In SCCs, 41 genes involved in cell cycle regulation were upregulated between 2 to 4.3-fold. Looking at key molecules for progression of cell cycle, the cyclines A2 and B2, cyclin-dependent kinase 4 and the cell division cycle 2 genes were upregulated. In the group of genes involved in DNA repair, we found genes with key functions for mismatch and double-strand DNA repair such as proliferating cell nuclear antigen, mutS homolog 6 replication factor C 4 and C5, RAD51 associated protein 1, which were overexpressed in comparison to normal lung tissue (Table 3).

**Table 3:** Selection of significantly differentially expressed genes in squamous cell carcinomas focusing on proliferation, cell structure and oncogenesis.

**Gene Symbol**	**Gene Name**	**Fold Change** **SCC vs. NT**	**q-value**
**Proliferation**			

RFC4	replication factor C (activator 4)	5.73	0.37

CCNB2	Cyclin B 2	4.34	0.37

PRC1	protein regulator of cytokinesis 1	3.54	1.21

CENPA	centromere protein A	3.29	0.61

MAD2L1	mitotic arrest deficient-like 1	3.20	0.61

CDK4	cyclin-dependent kinase 4	2.89	0.37

CDC2	cell division cycle 2	2.89	0.37

BUB1B	budding uninhibited by benzimidazoles 1 homolog beta	2.67	1.21

PCNA	proliferating cell nuclear antigen	2.62	0.37

PIR51	RAD51 associated protein 1	2.48	0.61

HEC1	kinetochore associated 2	2.15	0.84

MSH6	mutS homolog 6	2.10	1.06

RFC5	replication factor C (activator 5)	2.09	0.37

CCNA2	Cyclin A 2	2.05	1.06

CCNA1	Cyclin A 1	0.57	0.61

**cell structure**			

COL11A1	collagen, type XI, alpha 1	7.94	0.84

COL1A1	collagen, type I, alpha 1	3.24	1.21

TMSNB	thymosin, beta, 4X	3.24	2.53

COL5A2	collagen, type V, alpha 2	2.99	0.37

COL1A2	collagen, type I, alpha 2	2.89	0.84

PLS3	plastin 3	2.46	0.37

COL6A3	collagen, type VI, alpha 3	2.28	1.34

FSCN1	fascin, homolog 1	2.20	1.06

**oncogenesis**			

NMB	glycoprotein (transmembrane) nmb	4.01	1.34

RANBP1	RAN binding protein 1	3.23	0.37

MAF	v-maf Avian Musculoaponeurotic Fibrosarcoma oncogene	2.63	1.34

RACGAP1	Rac GTPase activating protein 1	2.53	0.61

PTTG1	pituitary tumor-transforming 1	2.49	1.34

MYC	v-myc Avian Myelocytomatosis viral oncogene homolog	2.43	1.90

RALA	v-ral simian leukemia viral oncogene homolog A	2.42	1.34

RAP2B	Ras-Related Protein 2B	2.30	0.84

RAN	Ras-Related Nuclear Protein	2.02	1.90

KIT	v-KIT Hardy-Zuckerman 4 Feline Sarcoma viral oncogene homolog	0.46	0.37

RABL2B	Rab-like 2B	0.35	0.37

RABL2A	Rab-like 2A	0.33	1.34

SCC = squamous cell carcinomas, NT = normal lung tissue

### Small cell lung cancer

In SCLC, we found 404 differential expressed genes, including 223 upregulated and 181 downregulated genes. Looking at oncogenes and tumor-associated genes, 4 genes of the rat sarcoma viral oncogene homolog associated gene family, FYN oncogene related to SRC and pituitary tumor-transforming 1 were upregulated, respectively. Of interest, the three tumor-related genes: tumor protein D52, melanoma antigen family D 4, stathmin 1/oncoprotein 18 and two oncogenes DEK oncogene and forkhead box G1 were upregulated which has not been described in the context of lung cancer so far.

In comparison to normal lung tissue, a different pattern of cell adhesion molecules was found in SCLC, showing 8 genes up- and 7 genes downregulated between 2 to 12.8-fold and 2.1 to 4.6-fold, respectively. In particular, the neural cell adhesion molecule 1 and the neuronal cell adhesion molecule, both members of the immunoglobulin superfamily, were overexpressed. Looking for genes involved in cell cycle regulation, we found 56 genes upregulated between 2.1 to 5.1-fold compared to normal lung tissue among them the key molecules for progression of cell cycle, the cyclines A2 and B2, cyclin-dependent kinase 4 and the cell division cycle 2 genes and cyclin E. The expression patterns of genes of the centromer/kinetochore complex and genes involved in DNA repair were similar to the expression patterns in SCC (Table 4).

**Table 4:** Selection of significantly differentially expressed genes in small cell lung cancer focusing on proliferation, oncogenesis and cell adhesion.

**Gene Symbol**	**Gene Name**	**Fold Change** **SCLC vs. NT**	**q-value**
**proliferation**			

RFC4	replication factor C (activator 4)	5.56	0.09

p16/CDKN2A	cyclin-dependent kinase inhibitor 2A	5.15	0.23

CENPA	centromere protein A	4.68	0.09

CCNE	Cyclin E	4.48	0.09

CCNB2	Cyclin B2	4.43	0.09

PRC1	protein regulator of cytokinesis 1	4.23	0.28

CENPF	centromere protein F	4.01	0.23

MAD2L1	mitotic arrest deficient-like 1	3.78	0.09

HEC1	kinetochore associated 2	3.66	0.09

PIR51	RAD51 associated protein1	3.60	0.09

BUB1B	budding uninhibited by benzimidazoles 1 homolog beta	3.51	0.09

CDC2	cell division cycle 2	3.42	0.28

PCNA	proliferating cell nuclear antigen	3.12	0.09

RFC3	replication factor C (activator 3)	2.58	0.09

RFC5	replication factor C (activator 5)	2.55	0.09

CDK4	cyclin-dependent kinase 4	2.55	0.09

NEK2	never in mitosis gene a-related kinase 2	2.48	0.09

CDK2	cyclin-dependent kinase 2	2.41	0.09

BUB1	budding uninhibited by benzimidazoles 1 homolog	2.38	0.09

CENPE	centromere protein E	2.38	0.09

ENC1	ectodermal-neural cortex 1	2.32	0.23

MSH2	mutS homolog 2	2.31	0.09

FANCG	Fanconi anemia, complementation G	2.22	0.09

MSH6	mutS homolog 6	2.09	0.09

CCNA2	Cyclin A2	2.07	0.09

**oncogenesis**			

FOXG1B	Forkhead BOX G1B, QIN oncogene	16.76	0.09

STMN1	stathmin 1/oncoprotein 18	4.98	0.16

FYN	fyn oncogene related to src, fgr, yes	3.91	0.16

MAGED4	melanoma antigen, family D, 4	3.51	0.28

PTTG1	pituitary tumor-transforming 1	2.56	0.09

RACGAP1	Rac GTPase activating protein 1	3.30	0.09

DEK	DEK oncogene (DNA binding)	2.49	0.16

RAN	Ras-Related Nuclear Protein	2.39	0.16

RANBP1	RAN binding protein 1	2.39	0.16

TPD52	tumor protein D52	2.08	0.16

RABL2B	Rab-like 2B	0.44	0.09

RABL2	Rab-like 2A	0.40	0.09

**Cell adhesion**			

NCAM1	neural cell adhesion molecule 1	12.75	0.16

NRCAM	neuronal cell adhesion molecule	9.43	0.09

CDH2	N-cadherin	5.41	0.09

CELSR3	cadherin, EGF LAG seven-pass G-type receptor 3	2.46	0.09

TJP3	tight junction protein 3, zona occludens 3	0.45	0.09

SCLC = small cell lung cancer, NT = normal lung tissue

### Corroboration of array data by quantitative real-time (RT) PCR

**Figure 3:**
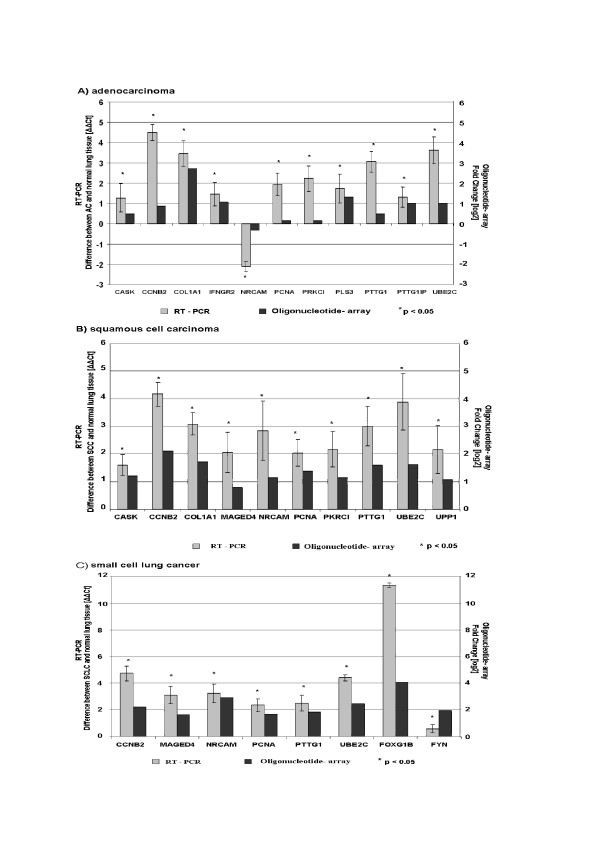
Corroboration of the results from microarray analysis using RT-PCR. The gene expression levels were normalized to a housekeeping gene (RPS11) for calculating ΔΔCt values. A ΔΔCt value of 1 corresponds to a Fold Change of 2. A) adenocarcinomas (AC), B) squamous cell carcinomas (SCC) and C) small cell lung cancer (SCLC).

Quantitative RT-PCR was used to verify the microarray data for 13 genes found to be differentially regulated in the different histologic lung cancer subgroups as compared to normal lung tissue. The 13 tested genes that were selected from different functional classes, with focus on the genes presented in tables 2–4, were: CASK (calcium/calmodulin-dependent serine protein kinase), CCNB2 (cyclin B2), COL1A1 (collagen, type I, alpha 1), IFNGR2 (interferon gamma receptor 2), PCNA (proliferating cell nuclear antigen), PRKCI (protein kinase C, iota), PLS3 (plastin 3), PTTG1 (pituitary tumor-transforming 1), PTTG1-IP (pituitary tumor-transforming 1 binding protein), UBE2C (ubiquitin-conjugating enzyme E2C), MAGED4 (melanoma antigen family D 4), FOX (forkhead box G1) and FYN (FYN oncogene related to SRC) and NRCAM (neuronal cell adhesion molecule). The expression data generated by the oligonucleotide array and RT-PCR were highly concordant, supporting the reliability of the array analysis (Figure 3). Of interest, the pituitary tumor-transforming gene 1 was 2.56 and 2.49-fold significantly differentially expressed in SCLC and SCC, respectively, in comparison to normal lung tissue using microarray analysis. In AC, the difference of expression was not significant in microarray analysis. However, using RT-PCR for corroboration, the pituitary tumor-transforming gene 1 was 5.7, 8.0 and 8.3 overexpressed in SCLC, SCC and AC, respectively, in comparison to normal lung tissue. In a previously conducted immunohistochemical study, we have demonstrated a strong pituitary tumor-transforming gene 1 expression in SCLC, adenocarcinomas, as well as in SCC, whilst a weak expression was only found in the luminal layer of normal lung epithelia, thus supporting the data of RT-PCR [27].

### Class prediction using genetic programming

In order to identify genes that enable accurate distinction between AC, SCC and SCLC, as well as normal lung tissue, a genetic programming data analysis was performed. The percentages of exact predictions for all samples of a class using 1020 classifiers (34 tissue samples and 30 classifiers = 34 * 30 = 1020 classifiers) are shown in Table 5 and the 10 genes with the highest frequency in each of the four classes were chosen in order to generate a final classifier of 40 genes. Using microarray training set of 34 samples (10 AC, 10 SCC, 9 SCLC and 5 normal lung tissues), a minimal set of 40 genes (Table 6) provided a classification accuracy for division into the 4 different cell tissues. For external validation, the test set included 13 different NSCLC samples from pretreated patients (9 recurrent AC and 4 recurrent SCC). All test set samples were correctly classified using the 40 genes found with genetic programming.

**Table 5:** Leave-one-out Cross Validation (LOOCV) accuracy for all one vs. rest experiments.

**Experiment**	**Accuracy of all 30 classifiers**
AC vs. rest	87.75%
SCLC vs. rest	93.92%
SCC vs. rest	82.25%
NT vs. rest	93.24%

**Table 6:** Genes found by genetic programming for discrimination between SCLC, NSCLC (AC and SCC) and normal lung tissue.

**Gene Symbol**	**Gene Name**	**Location**
**Discrimination AC vs. rest**		

CLCA2	chloride channel, calcium activated, family member 2	1p31-p22

EFS	embryonal Fyn-associated substrate	14q11.2-q12

FGG	fibrinogen, gamma polypeptide	4q28

GPCR5A	G protein-coupled receptor, family C, group 5, member A	12p13-p12.3

KRT7	keratin 7	12q12-q13

KRT5	keratin 5 (epidermolysis bullosa simplex)	12q12-q13

PTPRZ1	protein tyrosine phosphatase, receptor-type, Z polypeptide 1	7q31.3

SEMA3F	sema domain, immunoglobulin domain (Ig), (semaphorin) 3F	3p21.3

SERPINA1	serine (or cysteine) proteinase inhibitor, clade A member 1	14q32.1

SLC39A8	solute carrier family 39 (zinc transporter), member 8	4q22-q24

**Discrimination SCC vs. rest**		

ADCY3	adenylate cyclase 3	2p24-p22

ATP2B1	ATPase, Ca++ transporting, plasma membrane 1	12q21.3

CASK	calcium/calmodulin-dependent serine protein kinase	Xp11.4

CHST2	carbohydrate (N-acetylglucosamine-6-O) sulfotransferase 2	3q24| 7q31

DGKA	diacylglycerol kinase, alpha 80kDa	12q13.3

GOLPH2	golgi phosphoprotein 2	9q21.33

KIF13B	kinesin family member 13B	8p12

RAB17	RAB17, member RAS oncogene family	2q37.3

RAB40B	RAB40B, member RAS oncogene family	17q25.3

SCNN1A	sodium channel, nonvoltage-gated 1 alpha	12p13

**Discrimination SCLC vs. rest**		

CELSR3	cadherin, EGF LAG seven-pass G-type receptor 3	3p24.1-p21.2

CTSH	cathepsin H	15q24-q25

DLK1	delta-like 1 homolog (Drosophila)	14q32

ERBB2	v-erb-b2 erythroblastic leukemia viral oncogene homolog 2	17q11.2-q12

FANCA	Fanconi anemia, complementation group A	16q24.3

ID4	inhibitor of DNA binding 4, dominant helix-loop-helix protein	6p22-p21

ISL1	ISL1 transcription factor, LIM/homeodomain, (islet-1)	5q11.2

MGC13024	hypothetical protein MGC13024	16p11.2

POU4F1	POU domain, class 4, transcription factor 1	13q21.1-q22

XYLT2	xylosyltransferase II	17q21.3-17q22

**Discrimination NT vs. rest**		

ALOX15	arachidonate 15-lipoxygenase	17p13.3

ANKMY1	ankyrin repeat and MYND domain containing 1	2q37.3

C18orf43	chromosome 18 open reading frame 43	18p11.21

DNAI1	dynein, axonemal, intermediate polypeptide 1	9p21-p13

GSTA3	glutathione S-transferase A3	6p12.1

LRRC6	leucine rich repeat containing 6	8q24.22

MIPEP	mitochondrial intermediate peptidase	13q12

NKX3-1	NK3 transcription factor related, locus 1 (Drosophila)	8p21

RTDR1	rhabdoid tumor deletion region gene 1	22q11.2

VNN3	vanin 3	6q23-q24

AC = adenocarcinoma, SCC = squamous cell carcinoma, SCLC = small cell lung cancer, NT = normal lung tissue

## Discussion

In this study, a comparison of the expression pattern of the 3 major histological lung cancer subtypes, as measured by array analysis, is presented. In comparison to the normal lung tissue, 205, 335 and 404 genes in AC, SCC and SCLC were found to be at least 2-fold differentially expressed. Fourteen genes of different gene families were corroborated using RT-PCR.

In AC, we found an up-regulation of keratin 7, a characteristic finding for pathologists to diagnose this subtype of lung cancer. On the other hand, keratin 5 was downregulated in AC. The differential expression is already described as a separator between AC and SCC, in line with our results [28,29]. Looking at adhesion molecules in AC, a down-regulation of the desmosomes desmoglein 3 and desmocollin 3 was found. In this context, it was shown that the invasive behavior of cells is inhibited when transfected with desmosomal components [30], suggesting that down-regulation of the desmosomes in adenocarcinomas of the lung plays a role in the loss of cell to cell contact and tumor spreading.

The extracellular cell matrix receptors integrin alpha-3 and integrin beta-2 as well as the collagen binding protein-1 (SERPHINH1) were upregulated in AC. These genes have a high affinity to collagen IV and laminin, both essential components of the basement membrane [31], possibly mediating adhesion and invasion. Additionally, we found intercellular adhesion molecule 1 (ICAM1), a cell-adhesion molecule also binding to integrin beta-2 and promoting metastasis due to tumor cell adhesion to endothelium overexpressed in AC [32,33].

Looking at the oncogenes in SCC, we found genes of the RAS associated gene family, the myc myelocytomatosis viral oncogene homolog (MYC) and musculoaponeurotic fibrosarcoma oncogene (MAF) upregulated. MAF encodes for nuclear transcriptional regulating proteins with a leucine zipper motif, and was identified in the genome of the acute transforming avian retrovirus AS42, which induces fibrosarcomas and has the ability to transform chicken embryo fibroblasts [34].

It is noteworthy that in SCC 5 members of the collagen family type I, V, VI, and XI were upregulated. An increased collagen synthesis might be associated with carcinogenesis, as in patients with breast cancer the emerging fibrotic focus is regarded as an indicator of tumor angiogenesis and independent predictor of early metastasis [35].

SCLCs show an up-regulation of 3 proto-oncogenes, which have not been described in this context so far. The DEK oncogene encodes for a 375 amino acid chromatin binding protein, which introduces supercoiling in DNA. It has been described to be upregulated in other tumor types, such as bladder cancer, glioblastoma, melanoma and leukemia [36]. The Qin oncogene, originally isolated from avian sarcoma virus, causes oncogenic transformation. Qin is the avian orthologue of mammalian brain factor-1 or forkhead box G1 (FOXG1B), a gene which belongs to the human forkhead-box gene family [37]. Possibly related to the neuroendocrine differentiation of SCLC, forkehead box G1 is essential for the proliferation and survival of cerebro-cortical progenitor cells [38]. Further, we found the Fyn oncogene upregulated in SCLC. Fyn is a member of the src family which is activated in colorectal cancer, and has also been identified in melanoma cells with elevated cell motility and spreading ability [39,40].

With regard to adhesion molecules, the overexpressed neural cell adhesion molecule 1 is useful for the diagnosis of SCLC [41,42]. Next to neural cell adhesion molecule 1 we found other genes significantly upregulated such as the Purkinje cell protein 4, secretory granule neuroendocrine protein 1, synaptotagmin 1 and the neuronal cell adhesion molecule (NRCAM) that seems to reflect the neuronal heritage of this particular lung tumor subtype. NRCAM belongs to the L1 family immunoglobulin-like CAMs, which are involved in the guidance, growth and fasciculation of neuronal cells [43]. Neuronal cell adhesion molecule has also been described in 2006 by Taniwaki and colleagues', who performed comprehensive gene expression profiles of pure SCLC cells derived from laser-microdissected tissue samples [44]. In order to confirm the overexpression of the neuronal cell adhesion molecule using a different technique, we corroborated the result of microarray analysis using RT-PCR, showing a 9.3-fold overexpression of the neuronal cell adhesion molecule in SCLC in comparison to lung tissue.

The imbalance of activated oncogenes and lost tumor suppressor genes, found in different types of lung cancer, may be associated with the different tumor growth kinetics. SCLC is the fastest growing lung tumor with a median tumor doubling time of 50 days [45]. This is reflected by our data with regard to the number and strength of upregulated cell cycle genes affecting growth rate. Several cyclines, their associated cyclin-dependent kinases and cell division cycle (CDC) genes controlling cell cycle progression, such as cyclin A2, B2 and E2, and cyclin-dependent kinase 2 and 4, as well as cell division cycle 2, 20 and 25B were upregulated [46]. The activation level of different cell cycle genes may be relevant with regard to new antitumor agents, which selectively target cell cycle proteins. For example, flavopiridol has the ability to induce cell cycle arrest by binding and inhibiting different cyclin-dependent kinase such as 2 and 4 [47,48]. Both CDKs are significantly upregulated in SCLC. On the other hand, the upregulation of cyclin-dependent kinase 2, that is critical for cell entry and progression through S phase of the cell cycle, is missing in NSCLC. Preclinical data support this finding since most NSCLC cell lines are resistant to flavopiridol-induced apoptosis unless they were treated during S phase. Furthermore, the IC 50 of flavopiridol-treated cells in SCLC cell lines is three times lower compared to NSCLC cell lines [49]. Consequently, this drug might be more promising in patients with SCLC.

We have further shown that genes involved in mismatch repair, such as mutS homolog 2 or 6, were upregulated in SCLCs, which is in line with other reports showing that these gene transcripts and proteins are present [50,51], in contrast to NSCLCs, where high resolution deletion mapping reveals frequent allelic losses at the DNA mismatch repair loci mutS homolog 3 [52]. Similar to the latter report, we have observed a downregulation of mutS homolog 3 in ACs.

After outlining potentially important molecular differences in different subtypes of lung cancer to normal lung tissue, we were interested in defining how many and which genes are necessary for correct classification of the lung tumor subtype. Using genetic programming (GP), a training set of 34 tissue samples was applied. With an evolutionary algorithm of GP, 40 genes were sufficient for a correct discrimination between all lung tumor tissue types and normal lung tissue. The 40 selected genes, identified using GP, were a subset of the genes, which were previously identified to be differentially expressed using cluster analysis. Following identification of the 40 genes with GP, further 13 tissue samples of previously treated patients NSCLC lung cancers were correctly classified with 100% prediction accuracy. It is important to note that the samples of the training set were from treatment naïve patients, while the test set came from those that were previously treated for their cancer using platinum-based chemotherapy. Nevertheless, the presented genes for distinction seem to maintain their value, independent from whether or not the patient had been treated. However, caution must be applied, since in the test set did not contain additional SCLC samples and larger sample size is needed which includes samples from all lung cancer subtypes in order to confirm the predictor.

## Conclusion

Our data show the different gene expression profiles in dependence from the histological type of lung cancer, which reflects the specific biological characteristics of the respective tumor subtype. These data may form the basis for a molecular classification system and allows a further insight into the altered genomic progress of the lung cancer cell, which may help to develop molecularly targeted drugs.

## Competing interests

The authors declare that they have no competing interests.

## Authors' contributions

AR, UPR, GP, RH and RK were involved in the design and/or conduct of the experiments as, well as the preparation of the manuscript. HG and HEG were involved in the histopathological review of the tumor samples. AS was involved in the tumor sample collection. AVH and MR were involved in the statistical analysis of the data. JN, GG and MS were involved in the data collection of the patients, and in the review of the manuscript.
